# Pathophysiological Mechanisms of Premature Ventricular Complexes

**DOI:** 10.3389/fphys.2020.00406

**Published:** 2020-05-13

**Authors:** Mark G. Hoogendijk, Tamás Géczy, Sing-Chien Yap, Tamas Szili-Torok

**Affiliations:** Department of Cardiology, Erasmus MC, University Medical Center Rotterdam, Rotterdam, Netherlands

**Keywords:** premature ventricular complex, review, cardiomyopathy, pathophysiology, arrhythmia

## Abstract

Premature ventricular complexes (PVCs) are the most common ventricular arrhythmia. Despite the high prevalence, the cause of PVCs remains elusive in most patients. A better understanding of the underlying pathophysiological mechanism may help to steer future research. This review aims to provide an overview of the potential pathophysiological mechanisms of PVCs and their differentiation.

## Introduction

**FIGURE 1:**
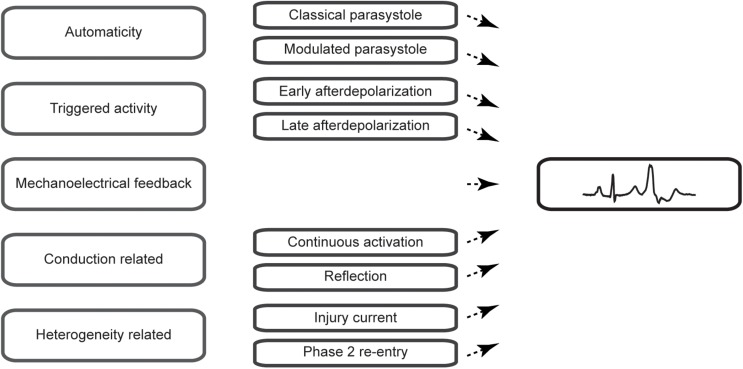
Schematic overview of the mechanisms of premature ventricular complexes covered in this review.

Premature ventricular complexes (PVCs) are the most common ventricular arrhythmia in healthy individuals and are frequently observed on ECGs (Hall et al., 1942) or during rhythm monitoring (Clarke et al., 1976). Although they may cause symptoms such as palpitations or dizziness, incidental PVCs are generally considered benign. Highly frequent PVCs, however, can have detrimental effects on the left ventricular function over time and may cause or worsen heart failure (Niwano et al., 2009; Baman et al., 2010). A reduction in their burden may, therefore, be beneficial. Indeed, observational studies have shown that a reduction of PVCs by the use of pharmacological agents such as β-blockers, amiodarone (Duffee et al., 1998) or flecainide (Hyman et al., 2018) or by catheter ablation (Takemoto et al., 2005; Yarlagadda et al., 2005) can improve the left ventricular function. Secondly, PVCs can serve as a trigger of idiopathic ventricular fibrillation and may present an ablation target to prevent recurrences of ventricular fibrillation (Haïssaguerre et al., 2002). Despite these insights in the potential clinical consequences, the cause of PVCs usually remains obscure as no practical methods exist to directly assess the pathophysiological mechanism in patients. In this review, we aim to provide an overview of the pathophysiological mechanisms of PVCs (Figure 1) and focus on characteristics that may aid in their differentiation.

## Automaticity

Automaticity in the sinus node underlies the normal rhythmic activation of the heart and is caused by spontaneous diastolic depolarization of specialized cardiac tissue [for an historic overview on the sinus node see Opthof (1988)]. It is, however, not a feature reserved for the sinus node and other structures, such as the Purkinje system, display diastolic depolarization (Draper and Weidmann, 1951). Automaticity outside the sinus node can cause PVCs via a mechanism called parasystole.

### Parasystole

Ectopic automaticity does not influence the cardiac rhythm under normal conditions as its diastolic depolarization is reset before reaching threshold potential by the activation of the faster sinus node. Enhancement of the automatic rate in the Purkinje by increasing the slope of diastolic depolarization system will result in a ventricular rhythm because the site with fastest automaticity determines the cardiac rhythm and, therefore, cannot explain the intermittent occurrence of PVCs. Other factors are required for automaticity to cause PVCs. These factors can be identified in the first experimental model of parasystole by Kaufman and Rothberger in which a stimulus timed by a metronome reflected an ectopic automatic focus (Kaufmann and Bothberger, 1917). First of all, the ectopic automatic focus needs to be protected from reset by the sinus rhythm without disabling its influence on the cardiac rhythm. Secondly, the cycle length of the sinus rhythm and the protected automatic ectopic focus need to allow for the ectopic beat to occur outside the ventricular refractory period. In this classical description of parasystole, PVCs occur at exact multiples of the cycle length of the ectopic automatic focus and can occur during the whole excitable period of the cardiac cycle (i.e., there is no fixed coupling interval of the PVCs).

### Modulated Parasystole

**FIGURE 2:**
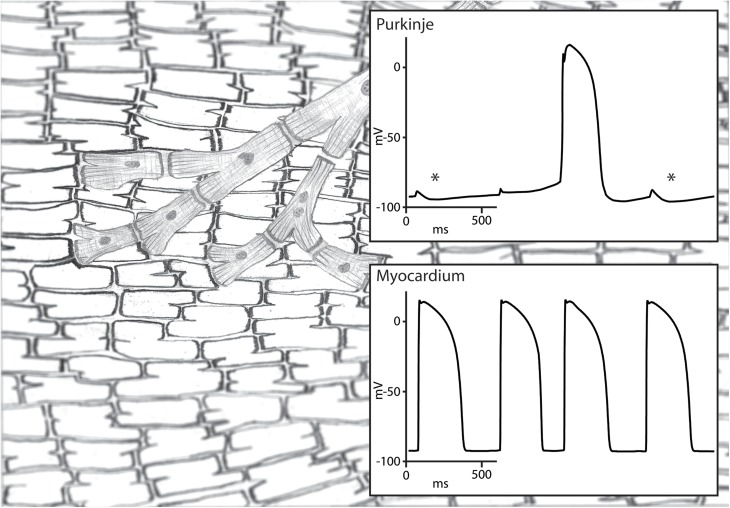
Illustration of modulated parasystole. A Purkinje fiber and ventricular myocardium are shown on the left and their transmembrane potentials on the right. The Purkinje fiber is protected from reset by the ventricular activation due to functional conduction block and is activated by its intrinsic automaticity. This automatic rhythm in the Purkinje fiber is influenced by the subthreshold depolarization by the surrounding myocardium. Subthreshold depolarization during the early phase 4 depolarization is followed by hyperpolarization (highlighted by an asterisk) which delays phase 4 depolarization reaching threshold potential. Subthreshold depolarization during later stage of phase 4 depolarization is not followed by hyperpolarization and advances the next automatic activation. The automatic activation of the Purkinje fiber activates the myocardium because the functional conduction block is unidirectional.

The conduction block that protects a parasystolic focus against excitation and reset has to be functional in nature. Otherwise, the parasystolic focus would not be able to conduct its activation to the myocardium and cause a PVCs. This functional block may still allow a degree of electrotonic interaction and may enable the modulation of a parasystolic focus by the activation front of the sinus rhythm (Jalife and Moe, 1976). Pivotal in the modulation of parasystole is the observation that subthreshold depolarization during diastolic depolarization influences the cycle length of automaticity in Purkinje fibers (Weidmann, 1951). In an elegant experimental setup, Jalife and Moe used isolated false tendons as a model of parasystole of which the center part was superfused with an isotonic sucrose solution resulting in local inactivation without cellular uncoupling. They demonstrated that activation on one side caused a subthreshold depolarization on the other side of the inactivated central area through electrotonic interaction. The influence of the subthreshold depolarization on the parasystolic cycle length depended on the timing during diastolic depolarization: early subthreshold depolarization delayed whereas late subthreshold depolarization advanced the next parasystolic activation (Jalife and Moe, 1976; Figure 2). This modulation makes the recognition parasystole more difficult because it alters the characteristic independent timing of extrasystoles seen in classical parasystole which will no longer occur at exact multiples of the parasystolic cycle length. What more, mathematical models have demonstrated that a high degree of modulation of the parasystolic cycle length causes PVCs to appear at preferential coupling intervals (Moe et al., 1977). This also hinders the identification of parasystole based on the spread of coupling intervals of the PVCs which are distributed evenly throughout the diastolic interval in classical parasystole. Nonetheless, a degree interdependence remains between the sinus rhythm and the occurrence of PVCs in modulated parasystole. More PVCs will occur at suitable ratios of cycle lengths of the sinus rhythm and the parasystolic focus (Moe et al., 1977). The demonstration of such suitable ratios of cycle lengths by altering one of these cycle length, for example by atrial stimulation at different frequencies, will provide support for the involvement of modulated parasystole.

## Triggered Activity

Automaticity is not the sole cellular mechanism for activation of the heart. Myocardial activations which characteristics were unlike the normal sinus rhythm were studied long before intracellular myocardial recordings could be used to study their causative mechanism. In contrast with automaticity, the activation does not occur spontaneously but requires a preceding action potential. Accounting for this prerequisite, these activations were termed triggered activity. Triggered activity is divided according to their timing in regards to the preceding action potential: the term early afterdepolarizations is used for reactivations during phase 2 or 3 whereas delayed afterdepolarizations occur after completion of the preceding action potential (Cranefield, 1975).

### Early Afterdepolarizations

Triggered activity has been studied in many models and under a wide variety of conditions. Valuable insight in the characteristics of early afterdepolarizations and the associated arrhythmias has been gained by the study of animal models from an organism to a cellular level. The first of these models was created by Brachmann et al. (1983) in an effort to study bradycardia-dependent triggered activity seen in patients with a prolonged QT-interval. To mimic these conditions they injected of formaldehyde into the atrioventricular node of dogs and infused cesium that further reduced the ventricular rate by suppressing automaticity and prolonged the QT-duration. This resulted in frequent PVCs and polymorphic ventricular tachycardias consistent with torsades de pointes. The importance of the heart rate was underscored by the demonstration of a relation between the duration of the polymorphic ventricular tachycardias and the duration of the preceding pause. Furthermore, both the extrasystoles and the tachycardias could be suppressed by increasing the heart rate through ventricular pacing. The underlying pathophysiological mechanism was studied further at a cellular level by exposing isolated canine Purkinje fibers to similar conditions by adding cesium to the superfusate and by using a low pacing frequency. The addition of cesium resulted in a prolongation of the action potential duration and in early afterdepolarizations leading to reactivations of the Purkinje fibers. Like in their *in vivo* experiment, increasing the pacing frequency successfully suppressed the arrhythmias.

Such a strict bradycardia-dependence of early afterdepolarizations has been challenged by studying the triggers of arrhythmias by early afterdepolarizations in congenital long-QT syndrome. Exercise which is usually accompanied with increased heart rates was the most common trigger (Schwartz et al., 2001) and no pause-dependent initiation of arrhythmic events could be demonstrated in patients with long-QT type 1 (Tan et al., 2006). Patients with long-QT type 1 carry a loss-of-function mutation in the gene *KCNQ1*, coding for the alpha subunit of the slow component of the delayed rectifier current (*I*_Ks_). β-adrenergic stimulation is known to increase the *I*_Ks_ and to cause a shortening of the action potential duration in isolated Guinea Pig cardiomyocytes (Sanguinetti et al., 1991). Beside affecting the *I*_Ks_, β-adrenergic stimulation also increases the L-type calcium current (*I*_CaL_) (Veldkamp et al., 2001) which being a depolarizing current prolongs the action potential. Presumably, loss-of-function mutations in *KCNQ1* reduce the ability to increase *I*_Ks_ and, thereby, to sufficiently counteract the effect of the increased *I*_CaL_ under conditions with an increased adrenergic tonus. This causes a prolonged QT-duration and predisposes patients with long-QT type 1 to arrhythmias by early afterdepolarizations during exercise.

### Delayed Afterdepolarizations

Pivotal in triggered activity by delayed afterdepolarizations is a transient inward current following an action potential that differs from the currents underlying the diastolic depolarization in automatic myocardial cells. This transient inward current is carried by the sodium-calcium exchanger (Fedida et al., 1987) that has electrogenic properties as it exchanges three sodium ions (Na^+^) with one calcium ion (Ca^2+^) over the cellular membrane (Blaustein and Russell, 1975). Under conditions of increased intracellular calcium concentrations, calcium is expelled from the cell through the sodium-calcium exchanger leading to a depolarizing current. The characteristics of delayed afterdepolarizations and the associated arrhythmias have been studied under many conditions leading to an increased calcium concentrations including digitalis toxicity, hypercalcemia, catecholamine exposure [for an overview see Wit and Janse (1993)] and altered cellular calcium handling through genetic mutations (Liu et al., 2006).

Some of the characteristics of delayed afterdepolarizations may be useful to differentiate the mechanism of PVCs in patients. The triggered activity by delayed afterdepolarizations usually occurs in bursts of reactivations. Interestingly, sustained ventricular tachycardias could be induced by exercise (Kim et al., 2007) and/or isoproterenol infusion (Yarlagadda et al., 2005; Kim et al., 2007) in a minority of patients undergoing ablation procedures for frequent PVCs from the ventricular outflow tracts. Additionally, changes in the coupling interval of PVCs may be studied. The degree of calcium loading does not only influence the amplitude but also the timing of the transient inward current underlying delayed afterdepolarization. This was demonstrated in isolated preparations of the canine coronary sinus during exposure to noradrenalin in which the calcium load was varied by changing the level of depolarization. Larger depolarization steps were followed by larger and earlier transient inward currents (Tseng and Wit, 1987). This would suggest that the coupling interval of PVCs should become shorter upon higher intracellular calcium concentration which can be altered by, for example, catecholamine exposure and/or by increasing the activation frequency. Lastly, the response to pacing differs between delayed afterdepolarizations and automaticity. Shorter coupling intervals of a single premature stimuli or shorter cycle length of a drive train were followed by larger delayed afterdepolarizations and ultimately resulted in triggered reactivations in simian mitral fibers exposed to catecholamines (*overdrive acceleration*) (Wit and Cranefield, 1976), whereas pacing of isolated canine and sheep Purkinje fibers in the absence of catecholamine exposure suppressed their automaticity in a rate and duration dependent matter (*overdrive suppression*) (Vassalle, 1970). Pivotal in this overdrive suppression of automaticity is the increased sodium entry into the cell at increased activation frequencies. Intracellular sodium can be exchanged for potassium via the sodium-potassium exchanger. As three sodium ions (Na^+^) are exchanged with two potassium ions (K^+^) this results in a hyperpolarization. This hyperpolarization slows the diastolic depolarization which delays the next automatic activation (Vassalle, 1970). Whether overdrive suppression is effective in PVCs by automaticity (parasystole) remains questionable as the parasystolic focus is protected from reset by the sinus rhythm. For effective overdrive suppression the activation front of the paced rhythm should either, in contrast with the sinus rhythm, be able to activate the parasystolic focus or the electrotonic interaction with the surrounding tissue should be sufficient for an effective hyperpolarization of the parasystolic focus.

## Mechanoelectrical Feedback

Mechanical strain has been implied to predispose patients to ventricular arrhythmias in various cardiac conditions including myocardial ischemia (Coronel et al., 2002), arrhythmogenic right ventricular cardiomyopathy (Fabritz et al., 2011) and cardiac arrest in patients with mitral valve prolapse (Sriram et al., 2013). This connection appears to be the clearest in survivors of cardiac arrest in association with mitral valve prolapse. These patients demonstrate frequent PVCs originating from the papillary muscle or left ventricular outflow tract region which are thought to be caused by the force of the mitral valve prolapse that is transmitted to the papillary muscle or by the diastolic contact between the mitral valve apparatus and the myocardium (Sriram et al., 2013). Additional support of the role of the mechanical stress in these patients have been found in histological and contrast-enhanced cardiac magnetic resonance imaging studies. Fibrosis in the papillary muscle and/or the inferobasal ventricular wall could be identified in sudden cardiac death victims with mitral valve prolapse as their only structural abnormality at autopsy. Likewise, late gadolinium myocardial enhancement as a sign of myocardial fibrosis was found in the same area in patients with mitral valve prolapse and ventricular arrhythmias from this region (Basso et al., 2015).

Sudden stretch of the myocardium has an electrophysiological effect on the myocardium dependent on the timing during the cardiac cycle (Zabel et al., 1996). A short stretch pulse during the plateau of the action potential causes temporary repolarization, whereas it causes depolarization and can cause a myocardial activation if applied during diastole. This mechanoelectrical feedback is attributed to the opening of stretch-activated ion channels on cardiomyocytes (Peyronnet et al., 2016). Usually, no mechanical stressor can be identified and an important role of mechanoelectrical feedback appears unlikely in most patients with frequent PVCs.

## Continuous Activation and Reflection

**FIGURE 3:**
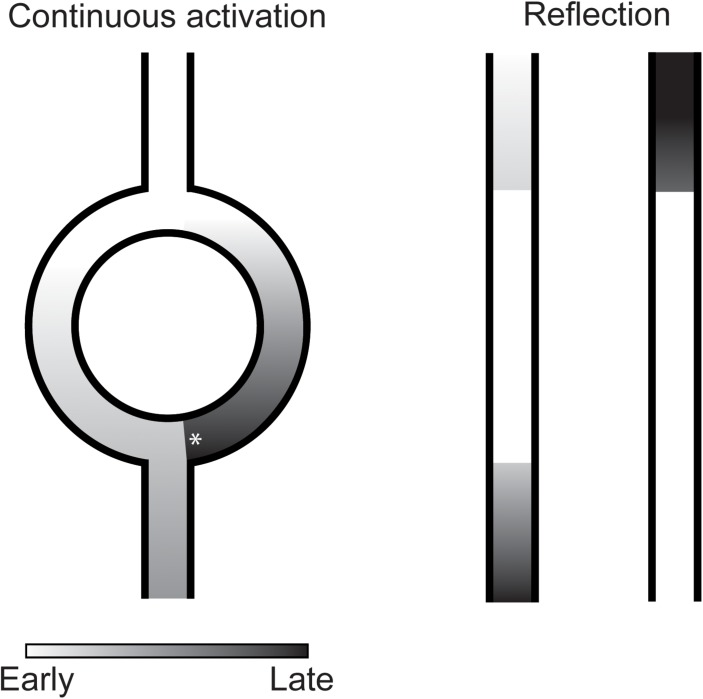
Illustration of continuous activation **(left)** and reflection **(right)** as mechanism of premature ventricular complexes. In continuous activation, a second activation front traveling through a separate pathway reactivates the myocardium. This separate pathway activates in only one direction which requires unidirectional block (asterisk) at the distal site. Furthermore, sufficient activation delay is required over this pathway to reactivate the myocardium after its refractory period. In reflection, activation delay over an inexcitable gap is sufficiently long for the myocardium to recover from refractoriness resulting in reactivation of the proximal myocardium.

Under special circumstances, a single sinus beat can activate the ventricles twice without the requirement of a cellular mechanism of reactivation. A well-known example of this is the double fire tachycardia observed in some patients with dual AV-nodal pathway physiology. In these patients, the activation front of a sinus beat travels through both a fast and a slow conducting pathway of the AV-node and activates the conduction system and ventricles twice. Similar conditions have been suggested to be present infranodal and to cause PVCs (Levy et al., 1975). In many reports, this mechanism is referred to as re-entry. Although, the mechanism shares several prerequisites of re-entry (the requirement of two separate pathways, unidirectional block in one pathway and sufficient activation delay over the other pathway to enable reactivation after the ventricular refractory period), the activation in both paths is in the same direction and the activation is not necessarily circular and, therefore, does not meet all the criteria of re-entry. In this paper we will refer to this mechanism of reactivation as continuous activation (Figure 3).

The demonstration of continuous activation can be challenging. The most direct indications of continuous activation causing PVCs were found during ablation procedures for ventricular tachycardias in ischemic cardiomyopathy. Low amplitude potentials preceded mapped PVCs that originated from scarred myocardium. These preceding potentials suggest the presence of a separate pathway supporting marked conduction delay required for PVCs by continuous activation. The overlap with re-entry was further illustrated by the fact that ablation aimed at PVCs also rendered the ventricular tachycardias non-inducible (Bogun et al., 2008). Theoretically, it may also be possible to demonstrate the continuous activation non-invasively using electrocardiographic signal averaging of the PVCs. This signal averaged ECG may visualize the continuous activation as a low-amplitude signal preceding a PVC.

Indications for another mechanism of reactivation depending on activation delay were found by Wit et al. (1972). They observed reactivations of unbranched Purkinje fibers in the opposite direction of the initial activation direction after excitability was depressed by hyperkalemia and after addition of l-epinephrine. What differentiated these reactivations from common re-entry was that the returning activation follows the same path as the antegrade conduction and, therefore, that no circuit could be identified (Wit et al., 1972). The mechanism at work, later termed reflection, was analyzed in more detail by local suppression of excitability of unbranched Purkinje fibers by regional encasement in hyperkalemic agar (Wit et al., 1972) or superfusion with an isotonic sucrose solution (Antzelevitch et al., 1980). The marked reduction in conduction velocity allowed the tissue proximal of the region with depressed excitability to be reactivated by the tissue distal through electrotonic interaction (Wit et al., 1972; Antzelevitch et al., 1980; Figure 3).

The mechanistic differentiation of reflection from modulated parasystole or continuous activation may be difficult clinically. First of all, the conditions supporting these mechanisms are quite similar with all them depending on localized functional conduction disturbances. As in the experimental models (Antzelevitch et al., 1980), multiple mechanisms may be operative in conjunction in individual patients. Moreover, these mechanism may demonstrate a similar response to physiological changes. For example, reflection in isolated Purkinje fibers appears to occur at favorable heart rates depending on the interplay between the activation frequency, conduction velocity and the recovery from refractoriness (Wit et al., 1972; Antzelevitch et al., 1980). Such a frequency dependency has also been suggested to occur in modulated parasystole (Moe et al., 1977).

## Heterogeneity: Injury Current and Phase 2 Re-Entry

Current flows driven by regional voltage gradients are integral to the normal myocardial activation and synchronization during the cardiac cycle. Altered current flows caused by abnormal voltage gradients during a susceptible window of the cardiac cycle may reactivate the heart.

Myocardial ischemia is one of the conditions in which such an altered current, the injury current, can reactivate the heart. The PVCs after ligation of the left anterior descending coronary artery were shown to originate from the myocardium bordering the ischemic zone early by Janse et al. (1980). Extracellular direct current electrograms demonstrated that that the injury current, caused by differences in resting membrane potentials and in repolarization time between the ischemic and non-ischemic myocardium, was usually enhanced just prior to the occurrence of PVCs. The calculated injury current was compared to currents calculated over a broad activation front after stimulation and considered of sufficient amplitude to underlie the observed PVCs (Janse et al., 1980). The injury current will, however, be an uncommon cause of frequent PVCs as it depends on the presence of myocardial ischemia which is unlikely to be present in most patients.

Abnormal voltage gradients in the absence of myocardial ischemia may also cause PVCs. As a theoretical proposition, Hoffman suggested as early as 1966 that marked local differences in the moment of repolarization may cause a current that can re-excite the myocardium with earliest repolarization that has had sufficient time for recovery of excitability (Hoffman, 1966). This mechanism was later termed phase 2 re-entry. Indeed, in tissue preparations with induced regional shortening of the action potential by potent sodium channel blockade by flecainide and increased pacing frequency (Krishnan and Antzelevitch, 1993) or by opening of the ATP-sensitive potassium current using pinacidil (Di Diego and Antzelevitch, 1993) reactivations have been observed at the sites with earliest repolarization that are consistent with phase 2 re-entry. The difficulty of inducing phase 2 re-entry can be found in whole porcine heart experiments in which sotalol, a *I*_Ks_ blocker, and pinacidil were added to the perfusate of adjacent coronary arteries aimed at reaching maximal repolarization gradients. Despite reaching an average repolarization gradient of 120 ms between these regions, no spontaneous arrhythmias occurred (Coronel et al., 2009). Such data suggest that either an even more severe provocation regimen or uncoupling may be required for reaching a sufficient repolarization gradient (Conrath et al., 2004). In patients, phase 2 reentry has been suggested to be the mechanism causing the PVCs that can initiate ventricular fibrillation in the Brugada syndrome (Yan and Antzelevitch, 1999). Direct epicardial assessment of the arrhythmic substrate, however, has since shown activation abnormalities in the form of low-voltage areas and fractionated electrograms (Nademanee et al., 2011) and are consistent with the notion that functional conduction disturbances and continuous activation cause the PVCs in Brugada syndrome (Hoogendijk et al., 2011). At least, phase 2 re-entry appears to be an uncommon mechanism in clinical practice as overt electrocardiographic abnormalities suggestive of the required excessive repolarization gradients are absent in most patients with frequent PVCs.

## Discussion

In this review, we provided an overview of the pathophysiological mechanisms of PVCs and focused on the features that may aid in their differentiation. An overlap in prerequisites and modulators exist which can make the differentiation of these mechanism difficult in clinical practice.

In an attempt to differentiate patients with idiopathic PVCs, (Bradfield et al., 2014) studied the variability of coupling intervals of PVCs in patients undergoing an ablation procedure. The variability was higher of PVCs originating from the sinus of Valsalva or great cardiac vein than of those from the right or left ventricle. Although interesting, no data are available to establish a cutoff to differentiate mechanisms according to the variability of coupling intervals of PVCs. It remains, therefore, unclear whether the findings reflect the involvement of a different mechanisms or of a different modulation of a single mechanism (e.g., various degrees of modulation of parasystolic foci).

A combination of observations have been used to study the cause of idiopathic PVCs from the ventricular outflow tracts by Kim et al. (2007) Sustained ventricular tachycardias with the same morphology as the PVCs could be induced by exercise in 10% and by isoproterenol infusion in 4% of patients (Kim et al., 2007). Following these observation, PVCs and idiopathic ventricular tachycardias from the outflow tracts were proposed to reflect a continuum of arrhythmias with delayed afterdepolarizations as their binding underlying cause (Kim et al., 2007). Although attractive, this hypothesis has some drawbacks. In experimental settings, the arrhythmias by delayed afterdepolarizations usually occur in bursts of reactivations. That only isolated PVCs were observed in most patients even upon provocation would, therefore, appear inconsistent with delayed afterdepolarizations being their underlying cause. Additionally, higher activation frequencies support delayed afterdepolarizations (Wit and Cranefield, 1976). Yet, no correlation or even an inverse correlation between the heart rate and occurrence of PVCs were found by the authors in most patients during holter monitoring (Kim et al., 2007). Considering these inconsistencies, it appears premature to interpret delayed afterdepolarizations as the sole cause of idiopathic outflow tract PVCs and different mechanisms may be involved.

In the studies mentioned above, the link between the location of origin and pathophysiological mechanism of a PVC was used as a premise. This premise, however, may not always be true. As mentioned before, different pathophysiological mechanisms can share the same prerequisites. The presence of a shared prerequisite can make a single anatomical location prone to develop PVCs via different mechanisms. The use of additional characteristics in that scenario may be required to further distinguish patients according to the etiology of their PVCs. For example, a feature that may help differentiate idiopathic PVCs by parasystole from delayed afterdepolarizations could be the presence of (non-)sustained ventricular tachycardias with the same morphology which cannot be explained solely by parasystole. Which characteristic is suited best for this further distinction is unknown and will depend on the assumptions on the pathophysiological mechanisms to be separated.

The recognition of the role of PVCs in the induction of heart failure (Niwano et al., 2009; Baman et al., 2010) or idiopathic ventricular fibrillation (Haïssaguerre et al., 2002) has renewed our interest in the cause of this common arrhythmias. We hope that from this new perspective, our work expands on previous reviews on this subject (Janse, 1992) and may serve as a framework for future thoughts on the pathophysiological mechanisms of PVCs.

## Author Contributions

MH wrote the manuscript. TG, S-CY, and TS-T reviewed and contributed to the final manuscript.

## Conflict of Interest

The authors declare that the research was conducted in the absence of any commercial or financial relationships that could be construed as a potential conflict of interest.
